# *In Silico* Identification and Comparative Genomics of Candidate Genes Involved in Biosynthesis and Accumulation of Seed Oil in Plants

**DOI:** 10.1155/2012/914843

**Published:** 2012-01-24

**Authors:** Arti Sharma, Rajinder Singh Chauhan

**Affiliations:** Department of Biotechnology & Bioinformatics, Jaypee University of Information Technology, Waknaghat, P.O. Dumehar Bani, Kandaghat, Solan 173 215, India

## Abstract

Genes involved in fatty acids biosynthesis, modification and oil body formation are expected to be conserved in structure and function in different plant species. However, significant differences in the composition of fatty acids and total oil contents in seeds have been observed in different plant species. Comparative genomics was performed on 261 genes involved in fatty acids biosynthesis, TAG synthesis, and oil bodies formation in Arabidopsis, *Brassica rapa*, castor bean and soybean. *In silico* expression analysis revealed that stearoyl desaturase, FatB, FAD2, oleosin and DGAT are highly abundant in seeds, thereby considered as ideal candidates for mining of favorable alleles in natural population. Gene structure analysis for major genes, ACCase, FatA, FatB, FAD2, FAD3 and DGAT, which are known to play crucial role in oil synthesis revealed that there are uncommon variations (SNPs and INDELs) which lead to varying content and composition of fatty acids in seed oil. The predicted variations can provide good targets for seed oil QTL identification, understanding the molecular mechanism of seed oil accumulation, and genetic modification to enhance seed oil yield in plants.

## 1. Introduction

A major challenge mankind is facing in this century is the gradual exhaustion of the fossil energy resources. The combustion of those fossil fuels used in transportation is one of the key factors responsible for global warming and environment pollution due to large-scale carbon dioxide emissions. Thus, alternative energy sources based on sustainable and ecologically friendly processes are urgently required. At present gasoline or diesel are being largely substituted by two biofuels, bioethanol and biodiesel, capturing *∼*90% of the market [1]. Biodiesel is made from renewable biomass mainly by alkali-catalysed transesterification of triacylglycerols (TAGs) from plant oils [2]. Manipulation of biosynthetic pathways offers a number of exciting opportunities for plant biologists to redesign plant metabolism toward production of specific TAGs.

The biosynthesis of fatty acids in plants begins with the formation of acetyl Co-A from pyruvate. The acetyl CoA produced in plastids is activated to malonyl CoA; the malonyl group is subsequently transferred to acyl carrier protein (ACP) giving rise to malonyl ACP, the primary substrate of the fatty acid synthase complex. The formation of malonyl CoA is the committed step in fatty acid synthesis and is catalyzed by the highly regulated plastidic acetyl CoA carboxylase complex [3]. *De novo* fatty acid synthesis in the plastids occurs through a repeated series of condensation, reduction, and dehydration reactions that add two carbon units derived from malonyl ACP to the elongating fatty acid chain. A series of condensation reactions proceed with acetyl-CoA and malonyl-ACP, then acyl-ACP acceptors. Three separate condensing enzymes, or 3-ketoacyl-ACP synthases (KAS I–III) are necessary for the production of an 18-carbon fatty acid. Three additional condensation reactions are required; each condensation step to obtain a saturated fatty acid that is two carbons longer than at the start of the cycle. These reactions are catalysed by 3-ketoacyl-ACP reductase (KAR), 3-hydroxyacyl-ACP dehydratase (HD), and enoyl-ACP reductase (ENR). The first desaturation step also occurs in the plastid; while the acyl chain is still conjugated to ACP, a Δ 9-desaturase converts stearoyl ACP to oleoyl ACP. Termination of fatty acid elongation is catalyzed by acyl ACP thioesterases, which are two main types in plants. The FatA class removes oleate from ACP, whereas FatB thioesterases are involved in saturated and unsaturated acyl ACPs, and, in some species, with shorter-chain-length acyl ACPs [4–6]. After release from ACP, the free fatty acids are exported from the plastid and converted to acyl CoAs. Nascent fatty acids can be incorporated into TAGs in developing seeds [4]. Oleic acid can be further desaturated to oleate acids by FAD2 [7] and FAD6 [8] in the cytosol and the plastid, respectively. Cytosolic and plastid *ω*-3 desaturations that result in the production of linolenic acids are catalyzed by FAD3 [9] and FAD7 [10], respectively. Fatty acids can be incorporated into TAGs in developing seeds in a number of ways. For example, a series of reactions known as the Kennedy pathway results in the esterification of two acyl chains from acyl CoA to glycerol-3-phosphate to form phosphatidic acid (PA) and, following phosphate removal, diacylglycerol (DAG). A diacylglycerol acyltransferase (DGAT), using acyl CoA as an acyl donor, converts DAG to TAG. Two classes of DGAT enzymes have been isolated [11, 12], and orthologs have been identified in numerous plant species. DAG and phosphatidylcholine (PC) are interchangeable via the action of cholinephosphotransferase, suggesting a route for the flux of fatty acids into and out of PC. Acyl chains from PC can be incorporated into TAG, either via conversion back to DAG or by the action of a phospholipid diacylglycerol acyltransferase (PDAT) that uses PC as an acyl donor to convert DAG to TAG. There are two predominant seed oil storage proteins in plants: caleosin and oleosin. TAG assembled in these storage proteins form oil bodies in seeds.

The fatty acid composition of seed oil varies considerably both between species and within species. The variation of fatty acids occurs both in chain length and degrees of desaturation. Consequently, the fuel properties of biodiesel derived from a mixture of fatty acids are dependent on the composition of fatty acids in seed oil. Altering the fatty acid profile can, therefore, improve fuel properties of biodiesel such as cold-temperature flow characteristics, oxidative stability, and NOx emissions [13].

Fatty acid biosynthetic pathway is highly conserved in plants, but there are significant variations in fatty acid contents and composition in plants (Table 1). What determines differences in the contents and composition of fatty acids and subsequently the total oil yield in the seeds is not understood. The availability of whole genome sequences, ESTs, and individual gene sequences from different oil rich plant species provide an opportunity to investigate what differences in the structure and sequences of genes determine variation in contents and composition so as to identify distinguishing gene signatures to assist in genetic improvement of crop plants either through marker-assisted breeding or by metabolic engineering [32]. Tanhuanpää et al. [33] developed an allele-specific PCR marker for oleic acid by comparing the wild-type and high-oleic allele of the FAD 2 gene locus in spring turnip rape (*Brassica rapa *ssp. *oleifera*). The accumulation of ricinoleic acid in transgenic Arabidopsis seeds was doubled by expressing the castor FAH12 hydroxylase in a FAD 2/FAE1 mutant [34]. The FatA and FatB genes of castor bean were heterologously expressed in *Escherichia coli* for biochemical characterization after purification, resulting in high catalytic efficiency of RcFatA on oleoyl-ACP and palmitoleoyl-ACP and high efficiencies of RcFatB for oleoyl-ACP and palmitoyl-ACP. The expression profile of these genes displayed the highest levels in expanding tissues that typically are very active in lipid biosynthesis such as developing seed endosperm and young expanding leaves [35]. *Arabidopsis thaliana* gene diacylglycerol acyltransferase (DGAT) coding for a key enzyme in TAG biosynthesis was expressed in tobacco under the control of a strong ribulose-biphosphate carboxylase small subunit promoter. This modification led up to a 20-fold increase in TAG accumulation in tobacco leaves and translated into an overall twofold increase in extracted fatty acids up to 5.8% of dry biomass in *Nicotiana tabacum* [36]. Dimov and Mollers [37] tested genetic variation for saturated fatty acid content in two sets of modern winter oilseed rape cultivars (*Brassica napus* L.) in field experiments under typical German growing conditions. They observed highly significant genetic differences among the cultivars for total saturated fatty acid content, which ranged from 6.8% to 8.1%. Singh et al. [38] constructed genetic map using AFLP, RFLP, and SSR markers for oil palm. They detected quantitative trait loci (QTLs) controlling oil quality (measured in terms of iodine value and fatty acid composition) and identified significant QTLs associated with iodine value (IV), myristic acid (C14 : 0), palmitic acid (C16 : 0), palmitoleic acid (C16 : 1), stearic acid (C18 : 0), oleic acid (C18 : 1), and linoleic acid (C18 : 2) content. The *Brassica napus* mutant line DMS100 carrying a G-to-A base substitution at the 5′ splice site of intron 6 in FAD 3 had reduced C18 : 3 content in oil seeds [39]. These studies suggest that the comparative analysis of oil biosynthesis and accumulation genes is a suitable strategy to investigate the molecular basis of oil content and composition variation in seed oils of different plant species. Additionally, these variations can be used to develop functional markers for increasing selection efficiency by marker- assisted selection in plant breeding.

In the present study, four plant species, Arabidopsis, Brassica, soybeans, and castor bean were considered for comprehensive analysis of fatty acid biosynthesis genes due to the availability of their genome sequences and several ESTs collections. Moreover, soybeans and brassicas are the biggest source of plant oil in the world, whereas castor bean contains unusual fatty acid ricinoleate that have chemical properties useful for industrial applications. The total seed oil contents of Arabidopsis, castor bean Brassica, and soybean are 30–37%, 40–45%, 30–40%, and 15–20%, respectively (Table 2) [28–31]. Plant oils are mostly composed of five common fatty acids, namely, palmitate (16 : 0), stearate (18 : 0), oleate (18 : 1), linoleate (18 : 2) and linolenate (18 : 3), although, depending on the particular species, longer or shorter fatty acids may also be major constituents. These fatty acids differ from each other in terms of acyl chain length and number of double bonds, leading to different physical properties. Here we put forward the questions (1) whether there are common variations in genes, if any, which contribute to increased seed oil content in plants? (2) Which are the major genes responsible for the higher amounts of five fatty acids mentioned above in different plant species? For answering these questions the present study aimed at (1) the identification of candidate genes for fatty acid biosynthesis, TAG synthesis and oil body formation proteins in plant species under study, (2) the comparative structure analysis of these candidate genes, (3) the *in silico* identification of sequence variations in fatty acid biosynthesis genes, and (4) the *in silico* association of sequence variations in candidate genes for oil content and composition.

## 2. Materials and Methods

### 2.1. Retrieval of Sequences

Thirty-two genes involved in the biosynthesis and storage of fatty acids were retrieved from Arabidopsis database (http://lipids.plantbiology.msu.edu/) by referring to the comprehensive lipid gene catalog provided by Beisson et al. [40]. The selected genes covered all the major biochemical events in the biosynthesis and storage of fatty acids [41, 42]. The protein sequences of these genes were used as query against castor bean database in TIGR (http://blast.jcvi.org/er-blast/index.cgi?project=rca1) and soybean database in soybase (http://soybase.org/). Full-length coding sequences of Brassica were downloaded from GenBank (http://www.ncbi.nlm.nih.gov/genbank/GenbankSearch.html). Protein function domains were examined with “CDD” from NCBI (http://www.ncbi.nlm.nih.gov/Structure/cdd/cdd.shtml).

### 2.2. Prediction of Gene Structures

Gene models for castor bean and soybean genomes were downloaded from Phytozome (http://www.phytozome.net/). The Arabidopsis gene models were downloaded from TAIR (http://www.arabidopsis.org/). Arabidopsis, castor bean, *Brassica. Rapa,* and soybean sequences were further annotated for gene models (open reading frames, including the 5′UTRs and 3′UTRs) using gene prediction algorithms of FGenesH (http://linux1.softberry.com/berry.phtml?topic=fgenesh&group=programs&subgroup=gfind) [43–45] (see Table 1 of the Supplementary Material available online at doi:10.1155/2012/914843). Sequence identity among *Brassica rapa*, castor bean, soybean, and Arabidopsis genes was confirmed using ClustalW in MegAling in DNASTAR (DNASTAR Inc., Madison, WI, USA). The *in silico* expression status of candidate genes belonging to different families was searched with an e-value cutoff 0.0 in the ESTdb of NCBI (National Centre for Biotechnology Information) at http://www.ncbi.nlm.nih.gov/BLAST/ and TIGR (The Institute of Genomic Research) at http://blast.jcvi.org/er-blast/index.cgi?project=rca1.

## 3. Results

### 3.1. Comparative Genomics of Fatty Acid Biosynthesis Genes in Major Oil Seed Plant Species

The fatty acid biosynthesis pathway includes 32 gene families involved in the conversion of acetyl Co-A into different fatty acids and their storage in oil bodies. A total of 68 protein sequences were retrieved for 32 gene families from the comprehensive lipid gene catalog of Arabidopsis [40] and functional domains were identified for each gene family. The 68 protein sequences from Arabidopsis were queried for fatty acid biosynthesis genes in *Brassica rapa*, soybean, and castor bean databases. A total of 261 genes belonging to 32 gene families were identified and retrieved from four plant species, out of which, 68 were from Arabidopsis, 62 from *Brassica rapa*, 55 from castor bean, and 76 from soybean (Table 3). Detailed gene structures, exon- intron coordinates of each gene are given in Supplementary Table  1.

### 3.2. Expression Status of Fatty Acid Biosynthesis Genes

**Figure 1:**
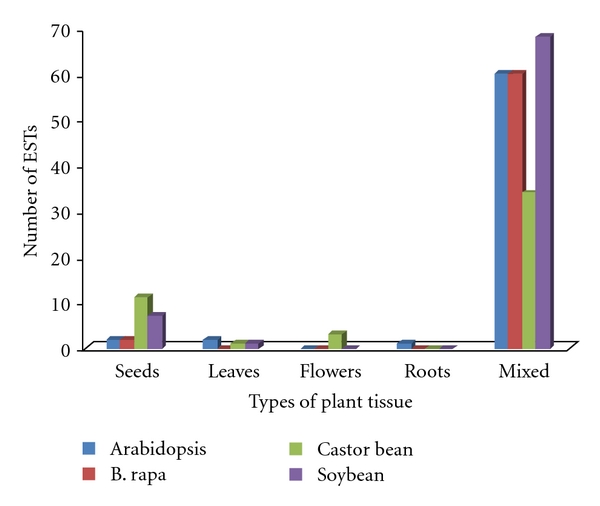
*In silico* transcript abundance (based on matching ESTs available in the database) of oil biosynthesis and accumulation genes in different tissues.

*In silico* expression analysis revealed that for 32 gene families, ESTs were detected for 68 genes in Arabidopsis, 62 genes in Brassica, 49 genes in castor bean, and 76 genes in soybean (Figure 1). Thirteen genes of Arabidopsis, 15 from castor bean, 8 from soybean, and 2 from Brassica showed tissue preferential expression patterns as per their identities to ESTs from tissue-specific libraries. Twenty-two genes from four plant species were expressed in seeds, 4 in leaves, 3 in flower, and 1 in roots (Table 4). FAD 2 and one homolog of Stearoyl desaturase gene had maximum seed ESTs in castor bean.

### 3.3. Comparative Analysis of Gene Structures in Different Plant Species

Comparative genomics of fatty acid biosynthesis genes was done to understand as what determines differences, if any, for variations in contents and compositions of fatty acids in different plant species. The gene structure analysis revealed that the exon-intron structure of fatty acid biosynthesis genes in castor bean and soybean gene homologs shared more structure similarity in comparison to Arabidopsis fatty acid biosynthesis genes. However, insertion, deletion, and intron size variations were found in castor bean and soybean genes with reference to Arabidopsis. Fatty acid biosynthesis genes of *Brassica rapa* were not analyzed for gene structure because for most of the Brassica genes only coding DNA sequences were available in the GeneBank.

**Figure 2:**
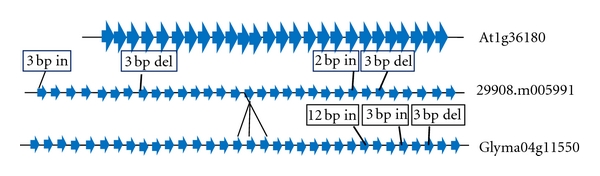
Structure of ACCase gene in Arabidopsis (26 exons), castor bean (31 exons), and soybean (33 exons); thick arrows and thin lines represented exons and introns, respectively. Arabidopsis 1–26 exons showed identity to 6 to 31st and 6 to 33rd exons of castor bean and soybean, respectively; 16th exon of castor bean showed identity to three exons of soybean (16th, 17th, and 18th). A 3 bp deletion (del) in the 8th and 26th exons of castor bean, 3 bp deletion and 3 bp insertion (in) in the 31st and 29th exons of soybean, and a 12 bp insertion in the 24th and 26th exons of castor bean and soybean, respectively. At1g36180: Arabidopsis ACCase gene; 29908.m005991: Castor bean ACCase gene; Glyma04g11550: Soybean ACCase gene.

Conversion of acetyl Co-A to malonyl Co-A by acetyl carboxylase (ACCase) is the most committed step in fatty acid biosynthesis. Exon/intron number and CDS length for ACCase gene was almost same between castor bean (31 exons) and soybean (33 exons), whereas slightly less in Arabidopsis (26 exons). Comparative structural analysis revealed that homomeric ACCase gene from Arabidopsis (1–26 exons) showed microsynteny with castor bean (6–31 exons) and soybean (6–33 exons), with a 3 bp deletion in 8th and 26th exons of castor bean, 3 bp deletion and 3 bp insertion in 29th and 31st exons of soybean, and a 12 bp insertion in 24th and 26th exons of castor bean and soybean, respectively. First five exons of homomeric ACCase in castor bean and soybean (missing in Arabidopsis) showed colinearity for exon size, with the exception of a 3 bp insertion in the first exon of castor bean gene. Sixteenth exon of ACCase in castor bean showed sequence identity to 3 exons (16th, 17th, and 18th) of soybean (Figure 2).

**Figure 3:**
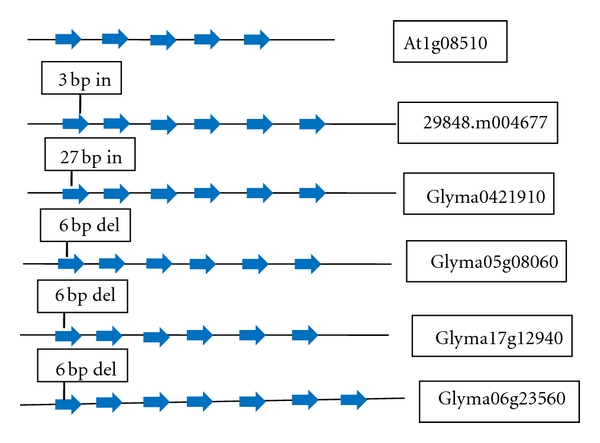
Structure of FatB (palmitoyl thioesterase) gene in Arabidopsis (At1g08510), castor bean (29848.m004677), and four soybean homologs (Glyma0421910, Glyma05g08060, Glyma17g12940, and Glyma06g23560). The 5th exon of FatB in Arabidopsis showed homology to the 6th exon of one of the homologs of soybean (Glyma04g21910) and last two exons (6th and 7th) of another homolog of soybean (Glyma06g23560), whereas 6th exon of castor bean showed homology to the 6th exons of other two homologs of soybean (Glyma05g08060 and Glyma17g12940).

Two distinct classes of thioesterases, FatA and FatB, are responsible for release of fatty acids from ACP by thioesterases. FatA gene structure was diverse with respect to exons number (varying from 5 to 11) among four plant species. Two homologs of FatA gene were present in Arabidopsis, castor bean, and soybean, whereas FatB gene had 4 homologs in soybean. The first exon of FatB gene had an insertion of 3 bp in castor bean and 27 bp insertion in one of soybean homologs (Glyma0421910) and other three homologs of soybean (Glyma05g08060, Glyma17g12940, and Glyma06g23560) had 6 bp deletion compared to Arabidopsis (Figure 3). An 69 bp insertion of one exon was present in FatB genes of castor bean and soybean but was absent in Arabidopsis. The last exon of FatB (5th exon) in Arabidopsis showed homology to the last exon (6th exon) of one of the homologs of soybean (Glyma04g21910) and last two exons (6th and 7th) of another homolog of soybean (Glyma06g23560), whereas last exon of castor bean showed homology to the last exon of other two homologs of soybean (Glyma05g08060 and Glyma17g12940).

**Figure 4:**
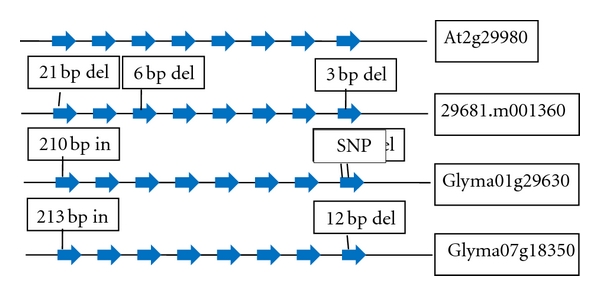
Structure of FAD 3 (linoleoyl desaturase) gene in Arabidopsis (At2g29980), castor bean (29681.m001360), and two soybean homologs (Glyma01g29630, Glyma07g18350). Exon/intron numbers are conserved in FAD 3 while variation in sizes was observed in the first and last exons. SNP identified in the 6th exon of soybean homolog (Glyma01g29630) was reported to be associated with low linolenic acid content [17].

Stearoyl ACP desaturase gene had maximum number of homologs (6 in Arabidopsis, 3 in Brassica, 4 in soybean, and 4 in castor bean) in fatty acid desaturase category of enzymes. Oleoyl desturase (Fad2) and Linoleate desaturase (Fad3) genes showed more relatedness in relation to number and sizes of exons and introns in each homolog among four plant species. Oleoyl desaturase (FAD 2) had only one exon in Arabidopsis, castor bean, and soybean with an insertion of 12 bp in the exon of castor bean and 9 bp insertion in the exon of one homolog of soybean (Glyma09g17170). FAD 3 gene structure was conserved with respect to exon-intron number and size between Arabidopsis, castor bean, and soybean except for first and last exons. A 21 bp deletion in the first exon of castor bean (29681.m001360) and an insertion of 210 and 213 bp was observed in two homologs of soybean (Glyma01g29630 and Glyma07g18350), respectively. Two deletions of 3 and 12 bp were observed in the last exon (8th exon) of castor bean and soybean, respectively. A deletion of 6 bp was observed in the 3rd exon of FAD 3 of castor bean. An SNP (G→A) was also identified at the exon-intron junction of FAD 3 gene in the 3rd exon of one homolog of soybean (Glyma01g29630) with respect to castor bean, Arabidopsis, and other homologs of soybean (Figure 4).

The DGAT gene involved in TAG (Tri-acyl Glyceride) synthesis has two isoforms, DGAT-1 and DGAT-2. These two genes showed variation in number and sizes of exons and introns. DGAT-1 gene had 15 exons in Arabidopsis, 13 exons in castor bean, and 16 exons in soybean. DGAT-2 had 8 exons in Arabidopsis and castor bean and 7 exons in soybean. The detailed comparative genomics of fatty acid biosynthesis genes in 4 oil seed plant species provided insights to undertake identification and utilization of castor bean fatty acid biosynthesis genes and sequence variations for the development of candidate gene markers in Jatropha.

Fatty acid biosynthesis genes showed evolutionary relatedness but there is no synteny in gene order and position of genes on the chromosomes. Location of genes on chromosomes in Arabidopsis and soybean is given in Supplementary Table 2. 

## 4. Discussion

In general, plant oil biosynthesis mostly follows the common biosynthetic pathways for fatty acids in the plastid as well as TAG in the endoplasmic reticulum (ER) and the oil further accumulates in oil bodies. However, there are significant differences for content and composition of seed oil in different plant species. Using comparative genomics, we tried to infer the effect of change in gene structure differences on oil content in different plant species. In this study, 261 genes involved in biosynthesis and accumulation of seed oil were identified in four oil seed plant species, Arabidopsis, Brassica, castor bean, and soybean. The genes corresponded to six different categories (ACCase, desturase, elongase, thioesterase, TAG synthesis and oil body proteins). Gene families corresponding to these six categories of enzymes had multiple copies in plant species with the exception of homomeric ACCase.

In higher plants, many proteins and enzymes are encoded by gene families, and in Arabidopsis, it has been estimated that 20% of genes are members of gene families [46]. The existence of gene families can sometimes reflect additional levels of genetic control or isoforms of proteins with specific functions. Therefore, it is of interest to detect potential gene families involved in the fatty acid biosynthesis pathway. There is a possibility that different copies of fatty acid biosynthesis genes are present in low oil content genotypes which gives leaky phenotypes as in the case of starch biosynthesis pathway where different copies of genes were responsible for low, medium, and high amylase contents in rice [47].

The oil biosynthesis may be limited by the production of fatty acids [48], which is regulated by acetyl CoA carboxylase (ACCase). Reduction of ACCase activity lowered (1.5–16%) the fatty acid content in transgenic seeds [49]. Conversion of acetyl Co-A to malonyl Co-A by acetyl carboxylase (ACCase) is the most committed step in fatty acid biosynthesis. ACCase of castor bean and soybean showed microsynteny to Arabidopsis, with a 3 bp deletion in 8th and 26th exons in castor bean, 3 bp deletion and 3 bp insertion in 29th and 31st exons in soybean and a 12 bp insertion in 24th, and 26th exons of castor bean and soybean, respectively with respect to Arabidopsis. These sequence variations in ACCase genes may be possibly influencing the variations in fatty acid composition and content in seed oil among Arabidopsis, castor bean, and soybean, as fatty acid content and composition was altered in many plant species with the variations in sequences or expression of ACCase gene [19, 50]. Yang et al. [19] identified two SNPs (T→G, G→A) in ACCase gene which lead to increase (1.3%) in oleic acid, lenolenic acid, and lenoleic acid content in maize. Addition of a plastid transit sequence targeted the introduced ACCase protein to chloroplasts, ultimately resulting in a 5% increase in seed oil of rapeseed [50]. The insertion or deletion identified in our analysis between Arabidopsis, castor bean, and soybean might be responsible for reduction or enhancement of ACCase activity, which is associated with the variations in total fatty acid composition in seed oil among these plant species.

Studies in transgenic plants have demonstrated that thioesterases contribute to the regulation of fatty acid chain length [51]. Typically, FatB accepts saturated acyl-ACP substrates of varying length, while FatA is specific to unsaturated fatty acids and acts on C18:1, oleic, acyl-ACPs [51]. In *Brassica napus *and Arabidopsis, genetic engineering of Acyl-ACP thioesterase (FatB) resulted in maximum increase of 58% in palmitic acid content [52, 53]. Preventing the release of saturated fatty acids from ACP by downregulating FatB, which encodes a palmitoyl ACP thioesterase, lowered the levels of saturated fatty acids [54]. Variations in palmitate content in seed oil in plant species can be related to the variations in FatB gene [27, 52, 53]. Cardinal et al. [27] identified deletion in exon-inrton junction in one homolg of FatB gene which was associated with low palmitic acid content in soybean cultivar Century (N79-2077 and N93-2008). Palmitate content was ~8% in Arabidopsis [55], ~2% in castor bean [56] and 7–11% in soybean [57]. Variations in the amount of palmitic acid in the seeds of Arabidopsis, castor bean, and soybean might be due to deletions in first exon of FatB gene, which can be further utilized for identification of markers associated with high level of palmitate (saturated fatty acid) in total seed oil in plant species desired for biodiesel purpose.

Soybean lines with high levels of oleic acid (85%) and low levels of saturated fatty acids (6%) have been developed using a transgenic strategy that results in downregulation of two genes, FAD 2, and FatB involved in fatty acid synthesis. Downregulation of the FAD 2 gene, encoding a Δ12 fatty acid desaturase, prevented the conversion of oleic acid to polyunsaturated fatty acids, resulting in increased levels of oleic acid. Additionally, preventing the release of saturated fatty acids from acyl carrier protein (ACP) by downregulating FatB gene, which encodes a palmitoyl ACP (acyl carrier protein) thioesterase, lowered the levels of saturated fatty acids [54]. Hu et al. [14] sequenced the FAD 2 gene fragment from the mutant line DMS100 and wild-type line Quantum of *Brassica napus*, and identified a single nucleotide mutation (C→T) in the FAD 2 gene. This particular mutation created a stop codon (TAG) leading to premature termination of the peptide chain during translation which leads to high oleic acid content in mutant line DMS100. *B. napus* mutant line DMS100 carrying a G-to-A substitution at the 5′ splice site of intron 6 in FAD 3 had reduced lenolenic acid content in seed oil [39]. In our analysis insertions or deletions in FAD 2 and FAD 3 genes of soybean might be the possible causes of higher oleate and linoleate content in high oil yielding soybean genotypes. Higher amount of ricinoleic acid in castor bean can be due to an insertion in the FAD 2 gene resulting in higher level of oleic acid because oleic acid is further utilized as a substrate by fatty acid hydroxylase (FAH) to convert oleate to ricinoleate. Low level of linoleate in castor bean oil may be due to a deletion in the 3rd exon of FAD 3 gene because each copy of FAD 3 in Arabidopsis and soybean is conserved.

In our analysis, the acyl-CoA:diacylglycerol acyltransferases (DGAT) gene was highly diverse, which might be involved in the overall variation in triacylglycerols in the oil among the plant species as it is a key enzyme in determining the levels of triacylglycerols in seed oils [58, 59]. Burgal et al. [58] demonstrated that coexpressing the castor bean DGAT2 gene with the castor FA 12 hydroxylase resulted in almost double the levels of hydroxylated fatty acids in neutral lipids (up to 30% of total, compared with 17% in the absence of DGAT2). In our study, most of the variations observed in the coding regions are either insertion or deletion of 3 bp or multiple of three that represent codon usage which either leads to shift in reading frame or functional mutation that are expected to be related to oil content. Thus, the sequence variations identified in fatty acid biosynthesis genes in this study can be tested for their functional role in altering content and composition of seed oil in Jatropha.

## 5. Conclusion

Comparative genomics, for gene structures and coding sequence variations, was performed on 261 genes involved in fatty acids biosynthesis, TAG synthesis, and oil bodies formation in four oil seed plant species, Arabidopsis, *Brassica rapa*, castor bean, and soybean to understand whether differences in gene structures or coding sequence determine preferential biosynthesis of higher amounts of particular fatty acids and their contents in the seeds of different plant species. Overall comparative gene structure of fatty acid biosynthesis related genes provided an insight to improve oil quality for biodiesel by exploiting the variations for engineering FAD5, FAD6, and FatB genes to enhance the content of saturated fatty acids. The variations in FAD2, FAD3, Stearoyl desaturase, DGAT-1, and DGAT-2 will be helpful to enhance the oil content in plants. The close relationship between genes under study would be helpful for comparative genomics to study these genes in related species for oil content modification.

## Figures and Tables

**Table 1:** Variations for fatty acids and TAG biosynthesis pathway genes associated with high oil content in different plant species.

Targeted Genes	Descriptions of variations	Gene regions harboring variations	Plant/organism	References
FAD 2, FAD 3	SNP for high oleic acid and low linolenic acid	Exon	Brassica	Hu et al., 2006 [14]
Stearoyl—ACP desaturase	SNP for high stearic acid	Exon	Soybean	Zhang et al., 2008 [15]
FAD 2	SNPs for high oleic acid	Exon	Peanut	López et al., 2000 [16]
FAD 3	SNP for low linolenic acid	Intron-Exon junction	Soybean	Bilyeu et al., 2005 [17]
KAS I	SNPs and Indel associated with oleic acid content	5′UTR, Exon, Intron	Soybean	Ha et al., 2010 [18]
KAS III, ACCase, Stearoyl—ACP desaturase, DGAT	Indels, SNPs and SSRs associated with variation in composition and concentration of oil	—	Maize	Yang et al., 2010 [19]
FAD 2	3 base pair variation leads to change in amino acid which contribute to high oleate content in oil	Exon	Peanut	Bruner et al., 2001 [20]
DGAT1	3 bp Insertion leads to high oleic acid content	Exon	Maize	Zheng et al., 2008 [21]
FAD 2	SSR linked to oleic acid content		Soybean	Bachlava et al., 2008 [22]
FAD 3	Deletion in soybean FAD 3 leads to reduced linolenate	Exon	Soybean	Anai et al., 2005 [23]
KAS III	SNP associated with high palmitic acid content	Exon	Soybean	Aghoram et al., 2006 [24]
Stearoyl—ACP desaturase	SSRs associated with high stearic acid	—	Soybean	Spencer et al., 2003 [25]
Stearoyl—ACP desaturase	SSRs and INDELs associated with high stearic acid	—	Sunflower	Pérez-Vich et al., 2006 [26]
FatB	Deletions associated with low palmitic acid content	Exons and Introns	Soybean	Cardinal et al., 2007 [27]

**Table 2:** Fatty acid composition of four plant species.

Fatty acid composition (%)	Arabidopsis [28]	Castor bean [29]	Brassica [30]	Soybean [31]
Palmitic acid	8.7	2.0	1.5	7–11
Stearic acid	3.6	1.0	0.4	2–6
Oleic acid	15.0	7.0	22.0	22–34
Linolenic acid	29.0	—	6.8	5–11
Linoleic acid	19.2	5.0	14.2	43–56
Ricinoleic acid	—	86–90	—	—
Others	24.5	—	47 (Erucic)	—

Total oil content	30–37	45–50	33–40	15–20

**Table 3:** Oil synthesis and accumulation genes in Arabidopsis, *Brassica rapa*, castor bean, and soybean.

Category	Gene name	Accession number				Coding DNA sequence length (bp)			
		Arabidopsis	*Brassica rapa*	Castor bean	Soybean	Arabid-opsis	*Brassica rapa*	Castor bean	Soybean
ACCase	ACCase	At1g36180	X77382	29908.m005991	Glyma04g11550	5997	2193	6723	6834
	Alpha-carboxyl transferase	At2g38040	AY538675	27798.m000585	Glyma18g42280	2346	2295	2355	2130
	Beta-carboxyl transferase	ATCG00500	Z50868	28890.m000006	—	636	984	528	—
	Biotin carboxylase	At5g35360	AY034410	30185.m000954	Glyma05g36450	1683	1608	1935	1731
	Biotin carrier	At5g15530	AY538674	29630.m000809 29929.m004560	Glyma08g03120	726	783	780924	1611

	Malonyl Co-A transacylase	At2g30200	AJ007046	30113.m001448	Glyma18g06500	1104	993	987	1113
	Beta-Ketoacyl ACP synthase I	At5g46290	AF244519	29693.m002034	Glyma10g04680	1422	1380	1437	1452
	Beta-Ketoacyl ACP synthase II	At1g74960	AF244520	29739.m003711	Glyma13g19010	1770	1302	2013	1305
	Beta-Ketoacyl ACP synthase III	At1g62640	AF179854	28455.m000368	Glyma09g41380 Glyma15g00550 Glyma18g44350	1215	186	1233	1194 831 1254
Elongase	3-Ketoacyl- acp- dehydrase	At1g62610 At3g46170 At3g55290 At3g55310	AF382146	30147.m013777	Glyma08g10760 Glyma18g01280	924 867 822 822		735	924 963
	3-Ketoacyl- Co-A reductase (KAR)	At1g24360	AY196197	29929.m004732	Glyma11g37320	927	960	987	963
	Enoyl-ACP reductase (ENR)	At2g05990	AJ243087 AJ243088 AJ243089 AJ243090 x95462	27843.m000160 29650.m000277	Glyma11g10770 Glyma12g03060 Glyma18g31780	11155	1158 1161 1161 1164 1158	1083 1083	1176 1203 1533
	Hydroxyacyl ACP Dehydrase (HD)	At2g22230	AF382146	30200.m000354	Glyma05g24650 Glyma08g07870 Glyma08g19200 Glyma15g05800	663	672	534	417 513 219 822
	Plastidial 1 acylglycerol phosphate acyltransferase	At4g30580	—	29687.m000572	Glyma06g28540 Glyma12g28470	1071	—	987	2406 1776
	Plastidial Glycerol phosphate acyltransferase	At1g32200	—	30068.m002660	Glyma09g34110	1380	—	1236	1413

	Monogalactosylacylglycerol desaturase (FAD 5)	At3g15850		29841.m002863	Glyma07g03370 Glyma08g22730	1116		1161	1101 1173
	Stearoyl-ACP desaturase	At1g43800 At2g43710 At3g02610 At3g02630 At3g02620 At5g16230 At5g16240	X63364 X74782 AY642537	27985.m000877 28470.m000428 29929.m004515 30020.m000203	Glyma02g15600 Glyma07g32850 Glyma13g08970 Glyma14g27990	1176 1206 984 1191 984 1206 1185	1200 1206 1200	1176 336 960 1191	1176 1176 1185 1014
	Oleate desaturase (FAD 6)	At4g30950	AY642535 AY642540	29696.m000105	Glyma02g36460	1347	1332 1293	825	1287
Desaturase	Linoleate desaturase (FAD 7)	At3g11170	AY592974 AY599884 FJ985689 FJ985690 FJ985691 L01418 L22962	28176.m000273 29681.m001360 29814.m000719	Glyma01g29630 Glyma03g07570 Glyma07g18350	1467	1320 1152 1134 1299 1335 1152 1134	1359 1131 1383	1359 1362 1362
	Linoleate desaturase (FAD 8)	At5g05580	AY592974 AY599884 FJ985691	28176.m000273 29681.m001360 29814.m000719	Glyma01g29630 Glyma03g07570 Glyma07g18350	1308 1152	1320 1152 1335	1359 1131 1383	1359 1362 1362
	ER-Oleate desaturase (FAD 2)	At3g12120	AY577313 DQ518276 DQ518277 DQ518278 FJ907397 FJ907398 FJ907399 FJ907400 FJ907401 FJ952144	28035.m000362	Glyma03g30070 Glyma09g17170 Glyma10g42470 Glyma19g32940 Glyma20g24530	1152	1155 780 780 780 1155 1155 1155 1155 1155 1155	1164	1152 1161 1140 1152 1140
	ER-Linoleate desaturase (FAD 3)	At2g29980	AY592974 AY599884 FJ985689 L01418 L22962	29681.m001360	Glyma01g29630 Glyma07g18350	1161	1320 1152 1134 1152 1134	1131	1359 1362

Thioesterase	Acyl-ACP thioesterase (FatA)	At3g25110 At4g13050	X87842	30217.m000262 29842.m003515	Glyma08g46360 Glyma18g36130	1089 1104	1176	1269 753	1191 1125
	Palmitoyl-ACP thioesterase (FatB)	At1g08510	DQ847275 Fj715952	29848.m004677	Glyma0421910 Glyma05g08060 Glyma06g23560 Glyma17g12940	1239	1245 1239	1260	1332 1251 1422 1251

TAG synthesis	Diacylglycerol Acyltranferase (DGAT 1)	At2g19450	AF164434	29912.m005373	Glyma13g16560	1593	1512	1830	1347
	Diacylglycerol Acyltranferase (DGAT 2)	At3g51520	AF155224	29682.m000581	Glyma09g32790	945	1056	768	987
	Lysophosphosphatidic acid acyltransferase (LPAAT)	At1g01610 At1g51260 At1g78690 At1g80950 At2g27090 At2g38110 At3g05510 At3g11430 At3g18850 At3g57650 At4g00400 At5g06090	AF111161 Gu045434 GU04535 Gu045436 Z49860	27810.m000646 29851.m002448 30169.m006433 30170.m013990 29736.m002070 29822.m003441 29969.m000267 30174.m008615	Glyma01g27900 Glyma03g01070 Glyma03g14180 Glyma07g07580 Glyma07g17720 Glyma10g23560 Glyma18g42580	1512 1119 873 1140 2232 1506 1347 1509 1146 1170 1503 1503	1035 1173 1176 1173 936 1188	1188 1050 852 738 1515 1539 594 1506	1536 678 858 675 1491 1428 1620
	Diacylglycerol cholinephosphotransferase	At3g25585	AY179560	30138.m003845	Glyma12g08720 Glyma0214210	1170	1449	1449	771 1188
	Digalactosyldiacyglycerol synthase (DGD1)	At3g11670	—	28726.m000069	Glyma03g36050 Glyma19g38720	2388	—	2538	2352 2361
	ER Phosphatidate Phosphatase	At1g15080	—	29586.m000620 29660.m000760 29660.m000759 29660.m000759	Glyma09g18450 Glyma10g41580 Glyma20g25650	813	—	954 564 930 945	957 969 909

Oil body protein	Caleosin	At1g23240 At1g23250 At1g70670 At1g70680 At2g33380 At4g26740 At5g55240	AY966447 DQ140380	29673.m000932 30008.m000820	Glyma3g41030 Glyma09g22310 Glyma09g22330 Glyma09g22580 Glyma09g25350 Glyma10g33350 Glyma19g43680 Glyma20g34300	669 663 588 552 711 738 732	717 705	597 702	723 615 606 384 384 570 723 402
	Oleosin	At1g48990 At2g25890 At3g01570 At3g18570 At3g27660 At4g25140 At5g40420 At5g51210	DQ328612 S37032	29794.m003372 30147.m013891 30147.m014333	Glyma05g07880 Glyma14g15020 Glyma17g13120	510 450 552 501 576 522 600 426	699 426	564 489 495	492 492 471

**Table 4:** *In silico* expression status of fatty acids biosynthesis and accumulation genes.

Tissue	Gene	Accession no.			
		Arabidopsis	*B. rapa *	Soybean	Castor bean
Seeds	Alpha carboxyltransferase				27798.m000585
	Enoyl ACP reductase				27843.m000160
	Stearoyl desaturase		X74782	Glyma13g08970	27985.m000877
	FAD-2			Glyma10g42470	28035.m000362
	ER Phosphatidate Phosphatase				29660.m000760
	DGAT 2			Glyma17g06120	29682.m000581
	FatB			Glyma17g12940	29842.m003515
	Oleosin		S37032	Glyma14g15020	30147.m014333
	Oleosin	At5g40420		Glyma17g13120	30147.m013891
	Oleosin				29794.m003372
	Hydroxyacyl ACP dehydrase				30200.m000354
	Caleosin	At5g55240		Glyma20g34300	

Leaves	FatB				29848.m004677
	LPAAT			Glyma07g07580	
	3-Ketoacyl- acp- dehydrase	At3g55290			
		At3g55310			

Flowers	DGD1				28726.m000069
	Beta- carboxyl transferase				28890.m000006
	ACCase				29908.m005991

Roots	Stearoyl desaturase	At3g02620			

Roots + flowers	Stearoyl desaturase	At3g02610			

Seed + flowers	Oleosin	At1g48990			

Leaves + flowers	FAD 7	At3g11170			
	Oleosin	At2g25890			
		At3g18570			
	Caleosin	At1g23240			
		At1g23250			
		At4g26740			
